# Advances in Polysaccharide-Based Oral Colon-Targeted Delivery Systems: The Journey So Far and the Road Ahead

**DOI:** 10.7759/cureus.33636

**Published:** 2023-01-11

**Authors:** Ibrahim M Ibrahim

**Affiliations:** 1 Department of Pharmacology, Faculty of Medicine, King Abdulaziz University, Jeddah, SAU

**Keywords:** colon-targeted drug delivery, oral drug delivery, dissolution methods, gut microbiota, colorectal diseases

## Abstract

Various colon-targeted oral delivery systems have been explored so far to treat colorectal diseases, including timed-release systems, prodrugs, pH-based polymer coatings, and microflora-triggered systems. Among them, the microbially triggered system has gained attention. Among various oral colon-targeted delivery systems discussed, the polysaccharide-based colon-targeted delivery system has been found to be quite promising as polysaccharides remain unaffected by gastric as well as upper intestine milieu and are only digested by colonic bacteria upon reaching the colon. The major bottleneck associated with this delivery is that non-suitability of this system during the diseased state due to decrease in bacterial count at that time. This causes the failure of delivery system to release the drug even at colonic site as the polysaccharide matrix/coat cannot be digested properly due to lack of bacteria. The co-administration of probiotics is reported to compensate for the bacterial loss besides facilitating site-specific release. However, this research is also limited at the preclinical level. Hence, efforts are required to make this technology scalable and clinically applicable. This article entails in detail various oral colon-targeted delivery systems prepared so far, as well as the limitations and benefits of polysaccharide-based oral colon-targeted delivery systems.

## Introduction and background

When it comes to gastrointestinal disorders, including ulcerative colitis, Crohn’s disease, and colon cancer, which are brought on by poor dietary and lifestyle choices, the oral route of administration is the most preferred [1]. Local delivery to the colon can improve the efficacy of medications used to treat colonic disorders [2].

In colon targeting, absorption of the formulations from the stomach and small intestine is minimized; hence, most of the administered dose reaches the lumen of the large intestine [3]. Furthermore, local drug delivery systems offer two key benefits, site-specificity and the release of the drug after a predetermined time interval [4]. Site-specific drug delivery stops the release of the drug in the upper part of the gastrointestinal tract (GIT), allowing the release of the drug in the colon. The release of the drug depends upon the transit time and different pH and microflora present in the GIT [5]. Moreover, colon targeting offers an opportunity for oral delivery of therapeutic proteins and peptides that otherwise would get degraded in the gastric milieu [6]. Such delayed processes are believed to improve a drug’s efficacy by concentrating the drug molecules where they are most needed, as well as by lowering the risks of adverse effects and drug toxicity associated with early drug release in the upper GIT. Targeted delivery to the colon would ensure direct treatment at the disease site, lower dosage, and reduced systemic adverse drug reactions [6].

The release of the drug is based upon the variations in the pH of GIT in the presence of diet, disease state, and consumption of food that affects the pH of GIT fluid. The shifts in pH along the GIT were used as a mechanism for the selective delivery of drugs to the colon. Another factor is the gastrointestinal transit time, which affects the movement of materials through the colon. The movement of materials is slow in the colon compared to other regions of the GIT. Variability in total transit time is typically influenced by several factors, including, but not limited to, food, particularly the amount of dietary fiber consumed, movement, stress, illness, and certain medications. Moreover, the colonic microflora can be utilized as a triggering factor for the drug release secondary to the degradation of the coating/protective layer used to target the drug to the colon and the breakdown of the linkage between the drug and the carrier [4].

## Review

The need for the oral colon-targeted drug delivery system

The colon-targeted drug delivery can offer several advantages over the conventional oral delivery system that include targeted delivery of drugs that tend to degrade in the stomach or small intestine; delivery of drugs that are poorly soluble; preventing drug absorption in the upper part of GIT avoiding first-pass metabolism; release of the drugs directly to the large bowel helping in dose reduction of cytotoxic drugs; better management of various ailments such as colorectal cancer, ulcerative colitis and Crohn’s disease with fewer side effects [7]; avoiding degradation of drugs in the gastric acidic environment, especially drugs that irritate the gastric mucosa; better patient adherence; and decrease in dosing frequency, which would help decrease the cost of expensive drugs [7].

Despite the aforementioned advantages, the oral colon-targeted drug delivery system (OCTDDS) also has certain drawbacks such as difficulties in delivering the drug to the distal colon; variation in the pH of GIT, and different enzymes interfering with the drug reaching their specific site; lesser availability of the drug at its specific site due to the presence of viscous colonic contents; difficult dissolution of poorly soluble drugs due to more viscosity of colonic content; the transport of the drug through the mucosa into the circulation is restricted because of the smaller surface area of the colon; and the physicochemical characteristics of the drug carrier also affect the targeted delivery system [8].

Approaches used for developing the oral colon-targeted drug delivery system

Various approaches that have been utilized so far for developing OCTDDS are based on the development of prodrugs, varying the pH, or using natural polysaccharides. The widely explored approaches can be categorized as primary approaches that include coating with pH-sensitive polymers, delayed-release OCTDDS, and microbially triggered OCTDDS. The latter utilizes various methods such as the synthesis of prodrugs, azo-polymeric prodrugs, and polysaccharide-based delivery systems. Furthermore, more advanced approaches for OCTDDS have been developed recently that include pressure-controlled OCTDDS, novel colon drug delivery system CODES™, and osmotic-controlled drug delivery [6].

To date, only pH-based OCTDDS have reached the market and none has been claimed as colon-targeted formulations. They are available in the form of delayed release. The seven formulations that are available in the market are listed in Table 1.

**Table 1:** Marketed products available for colon-specific drug delivery system.

Drug	Brand name	Dosage form	References
Prednisone	Deltasone	Enteric-coated tablets	Kesisoglou et al. 2005 [9]
Mesalamine	Asacol, Salofac, Claversal	Enteric-coated tablets	Sninsky et al. 1991; Friend 2005 [10,11]
Cyclosporine A	Sandimmune Neoral	Capsules	Zhang et al. 2010 [12]
Balsalazide	Colazal	Capsules	Edsbacker and Andersson 2004 [13]
Budesonide	Entocort	Enteric-coated tablets	Edsbacker and Andersson 2004 [13]
Beclomethasone	Clipper	Coated tablets	Rizello et al. 2002 [14]
Sulfasalazine	Azulfidine	Enteric-coated tablets	Nicholas et al. 2011 [15]

Polysaccharide-based drug delivery systems

Polysaccharides are monosaccharide-(sugar)-polymers that are abundant, widely available, affordable, and characterized by a wide range of characteristics and architectures. These can be chemically and biochemically changed and are extremely stable, healthy, non-toxic, hydrophilic, gel-forming, and biodegradable, indicating their usage in OCTDDS [16]. Numerous polysaccharides, including chitosan, pectin, chondroitin sulfate, dextrans, guar gum, inulin, pectin, cyclodextrins, locust bean gum, and amylose, have been evaluated as colon-specific drug carriers [17,18]. Various polysaccharides used for formulating OCTDDSs are listed in Table 2, along with their benefits and limitations. The most promising colon-specific drug delivery techniques are based on the enzymatic activity of colonic bacteria on polysaccharides [16].

**Table 2:** Various polysaccharides used in OCTDDS. OCTDDS = oral colon-targeted drug delivery system; GIT = gastrointestinal tract

Polysaccharide	Description	Advantages	Disadvantages	Reference
Chitosan	Source: Chitin Structural unit: β-(1/4)-linked D-glucosamine and N-acetyl-D-glucosamine units	Chitosan protects the drug from the cruel environment of the upper GIT and leads to the degradation of drug colonic enzymes. Chitosan is non-toxic, enzymatically degradable, and has mucoadhesive property	It is soluble at low pH, and it may lead to premature drug release in the stomach and small intestine	Hejazi and Amiji 2003; Umadevi et al. 2010; Ballarin-Gonzalez et al. 2013 [19-21]
Dextran	Source: Microbial origin Structural unit: a-(1/6)-linked D-glucose units with some degree of branching via a-(1/2), a-(1/3) and/or a-(1/4) linkages	Low cytotoxicity, biocompatible, biodegradable, and has anticoagulant property	Early drug release	Suflet et al. 2010; Sery and Hehre 1956 [22,23]
Pectin	Source: Dried citrus peel Structural unit: (1/4)-linked a-D-galacturonic acid	Gastric resistant and enzymatically degradable in the colon. Pectin act as a stabilizer, adsorbent, gelling, thickening, and bulk-forming agent	Higher water solubility. Early drug release	Wong et al. 2011 [24]
Hyaluronic acid	Source: Animal-umbilical tissue, and synovial fluid Microbial: Bacillus subtilis Fermentation Structural unit: D-glucuronic acid and N-acetyl- D-glucosamine linked by β-(1/3) linkages	Biocompatible, low immunogenicity, and undergoes enzymatic degradation in the colon. Produces therapeutic activity in intestinal inflammation	Allergic reactions. Early drug release	Heinze et al. 2012 [25]
Guar gum	Source: Seeds of Cyamopsis tetragonolobus Structural unit: (1-4) β-D manopyranosyl units with α-D galactopyranosyl units attached by (1-6) linkages	Better gelling and swelling properties. Retard the drug release in the upper GIT and susceptible to enzymatic degradation of the drug in the colonic region	Premature drug release.	Kumar et al. 2011 [26]
Inulin	Source: Polysaccharide obtained from family Compositae Structural unit: fructosyl groups linked by β-2,1 glycosidic bond	Protect the drug from the acidic environment in the stomach and provide the probiotic effect in presence of gut microflora	Indigestible in humans	Shah et al. 2011 [27]
Alginate	Source: Brown seaweeds Structural unit: β-D-mannuronic acid and α-L-guluronic acid linked with 1,4 glycosidic bonds	Biocompatible, bioadhesive, and biodegradable. Used in the formulation of hydrogels	Higher aqueous solubility	Shah et al. 2011 [27]
Chondroitin sulfate	Source: Cartilage Structural unit: D-glucuronic acid and N-acetyl D-galactosamine linked by β-1,3 glycosidic linkages	Biocompatible and biodegradable	Readily water soluble	Ramasamy et al. 2012 [28]
Ethylcellulose	Source: Polysaccharides obtained from cellulose Structural unit: Repeating anhydroglucose units	It slows the release of the drug and is used in sustained drug release	It inhibits drug release at higher concentrations. At lower temperatures, it becomes tacky	Rekhi and Jambhekar 1995 [29]

**Figure 1:**
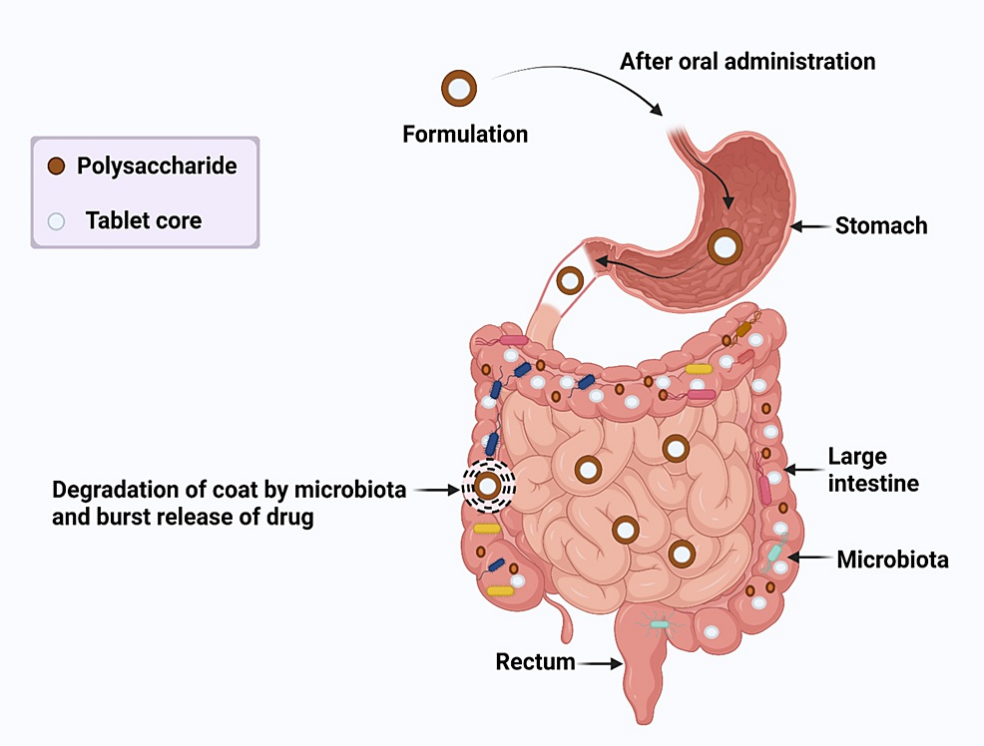
Mechanism of polysaccharide-based drug delivery. Kaushik et al. [30]. The figure was created by the author.

This mechanism is based on the breakdown of polymers covering the drug by microorganisms present in the colon system, resulting in drug load release in the colonic region. Intriguingly, the human GIT environment is characterized by the presence of complex microflora, notably in the microorganism-rich colon [30]. The relevant mechanism is depicted in Figure 1. There are numerous types of bacteria in colonic microflora that breakdown polysaccharides in the gut, including *Lactobacillus acidophilus*, *Lactobacillus GG*, *Lactobacillus casei Shirota*, *Streptococcus thermophiles*, *Bifidobacterium bifidum*, *Lactobacillus gasseri*, and *Lactobacillus reuteri* [31-34].

Case studies related to the development of polysaccharide-based delivery systems

Polysaccharide pectin-based colon-targeted delivery methods have been investigated. Calcium pectinate gel (CPG) beads were made by extruding bovine serum albumin (BSA)-loaded pectin solution into a calcium chloride solution that was being agitated. The release of BSA from CPG beads was investigated in vitro under conditions analogous to those in vivo. The type of pectin and the presence of an enzyme in the media affect the properties of release. By altering the kind of pectin, BSA can be protected against conditions prevailing from the mouth to the colon, as well as from enzymatic attack. In addition, the release of BSA from CPG beads was significantly altered by cross-linking duration but not by the amount of additional protein. In addition to influencing the bead sizes and entrapment efficiency, the analyzed variables also affected the size of the beads. Results suggested that CPG beads may be a suitable vehicle for colonic protein delivery [35].

Guar gum prevents the drug from being entirely released in the physiological milieu of the stomach and the small intestine, according to drug release tests that simulated the passage of food from the mouth to the colon. The vulnerability of guar gum to the colonic bacterial enzyme action and the subsequent release of indomethacin was proven by research conducted in phosphate buffer saline (PBS) with a pH of 6.8 that contained the caecal contents of rats. Inducing enzymes that only work on guar gum by pretreating rats orally with 1 mL of a 2% (w/v) aqueous dispersion of guar gum for three days led to an increase in the amount of medication that was released. After seven days of pretreatment, the caecal contents of rats showed a further rise in drug release compared to the beginning of the experiment. After three days and seven days of enzyme induction, biphasic drug release curves were seen in the presence of 4% (w/v) caecal content. The findings demonstrated that guar gum could serve as a viable carrier for the delivery of drugs with a specific target in the colon. The research also found that the optimal circumstances for in-vitro testing of guar gum are achieved by using 4% (w/v) of rat caecal contents in PBS, which is produced after seven days of enzyme induction. This combination of factors produced the most accurate results [36].

The drug delivery method of controlled release for indomethacin consisted of first dispersing the indomethacin in a solution and then dropping the solution containing the dispersion into a solution of calcium chloride. Ionotropic gelation caused the droplets to immediately coalesce into spheres that were gelled. On the percentage of drug entrapped, size distribution, and drug release from CPG beads, the effect of a few different elements such as the kind of pectin, the presence of a hardening agent, and the drug loading was explored. The rotating basket dissolution method was utilized for analyzing the release properties. In conclusion, it was possible to manufacture robust spherical beads with narrow size distributions, large yields, and excellent entrapment efficiencies [37].

The use of rofecoxib in the treatment and prevention of colorectal cancer was described by AI-Saidan et al. Wet granulation was used to create rofecoxib matrix tablets using guar gum as the binder. In-vitro and in-vivo drug release experiments were used to assess the effectiveness of the tablets. According to the findings of the in-vitro dissolution study, the rofecoxib tablets released between 5% and 12% of the drug in the upper GIT, whereas the tablet that contained 70% guar gum released 57% of the drug in the colon. This finding demonstrated that guar gum formulations were susceptible to the contents of rat caecal contents. During in-vivo testing with human volunteers, rofecoxib was observed to have properties that prevented the medication from being absorbed in the upper GIT. These properties included a delayed T_max_, lowered C_max_, and decreased ka in addition to a prolonged absorption period. The medication was transported to the colon, which led to a gradual increase in the rate of drug absorption and increased availability of the drug at the site of the human colon to cause the desired local impact [38].

The polysaccharide is administered to patients suffering from amoebiasis, a disease that is brought on by the parasite *Entamoeba histolytica* and affects the large intestine. In the treatment of amoebiasis and other anaerobic infections, the use of metronidazole and tinidazole is most commonly recommended. In this investigation, tablets of metronidazole were made with the help of xantham gum, guar gum, pectin, and carrageenan, which were then tested. In addition, to improve the targeting of the colon, the tablets were coated with Eudragit-L 100, which decreased the amount of drug released into the acidic environment by 18-24%. Xanthan gum polysaccharide, which was used as a matrix in the experiment, demonstrated time-dependent release characteristics. According to the findings of the dissolution study, the presence of rat caecal content led to an increase in the rate of drug release that was caused by polymer solubilization [39].

Krishnaiah and colleagues developed a drug delivery system for mebendazole that targets the colon. They did this by employing guar gum as a carrier. The method of wet granulation with starch paste serving as the binder was utilized in the preparation of matrix tablets. These tablets include varying concentrations of guar gum. In-vitro dissolution testing was performed on the formulation that had been developed, and high-performance liquid chromatography (HPLC) was used to determine the amount of medication that was released. In the presence of rat caecal content, the formulation that contained 20% guar gum released 80% of the drug, whereas another formulation that contained 30% guar gum released 50% of the drug in the colonic fluid [40].

Compression-coated, fast-disintegrating tablets of 5-fluorouracil (5-FU) using varying ratios (60%, 70%, and 80%) of guar gum were prepared and reported by Krishaniah et al. (2002). The in-vitro dissolution studies indicated a maximum of 4% drug release in gastric fluid. Upon subjecting the tablet to a medium containing rat caecal contents (4% w/v) after five hours, the tablets coated with 60%, 70%, and 80% guar gum showed 70%, 55%, and 41% drug release, respectively. Hence, the authors concluded that the tablet containing 80% guar gum was best for local action to target the colon [41].

Guar gum-based multilayer colon-targeted tablets of metronidazole were prepared by compression coating and compared with its matrix tablet. About 50% of drug release was observed from the matrix tablet during the dissolution study, whereas 25-44% of the drug was released from multilayer tablets in the stomach, followed by the small intestine. However, the multilayer compression-coated tablet released only 1% metronidazole in the first five hours, indicating the potential of the developed multilayered, compression-coated, guar gum-based formulation to target the colon. The rest of the drug got released in a sustained manner after five hours, indicating local and sustained release of the drug at the colonic site [42].

In a study by Raghavan et al., colon-targeted mesalazine tablets were formulated and evaluated using polysaccharides locust bean gum and chitosan. It protected the drug from the acidic environment of the upper GIT. The in-vitro study revealed that the maximum drug release occurred after five hours due to enzymatic degradation of the coating material in the presence of pH 6.8-PBS with or without rat caecal content. The in-vivo study stated that the pharmacokinetic parameters of the formulation demonstrated drug release after five hours [43].

A clinical cross-over study was performed for metronidazole (500 mg) loaded in immediate-release and guar-based colon-targeted tablets. The study showed that the immediate appearance of C_max_ was delayed by nine hours (T_max_) in the case of metronidazole loaded in colon-targeted tablets upon oral administration. This indicated the potential of guar gum in retarding the drug release in the upper GIT, i.e., the stomach and small intestine, and complete delivery of the drug upon reaching the colon. Moreover, the C_max_ that appeared in the colon was 25.76 mg/mL, and the C_max_ that appeared for the immediate-release tablet was 37.266 mg/mL, indicating poor absorption of the drug from the colonic site, thus making the drug effective for local action [44].

Calcium pectinate was a component of the formulation process for the enteric-coated theophylline microspheres. It was explored if the kind of pectin (amidated or non-amidated) and the preparation circumstances for the microspheres (CaCl_2_ concentrations and the type of cross-linking) had an effect on the amount of drug that was entrapped and how it was released. The coating not only made the microspheres more stable during storage but also prevented the morphologic alterations that were seen when uncoated microspheres were kept in conditions similar to those found in the environment. The length of time required for cross-linking was cut down, and the concentration of CaCl_2_ was increased (but only in the case of non-amidated pectin). This led to an increase in entrapment efficiency. On the other hand, a release test that was performed by simulating the pH variation in the GIT found an inverse relationship between the concentration of CaCl_2_ and the rate at which the drug was released, while pectin and the amount of time it was cross-linked were found to have no influence. The addition of pectinolytic enzymes to the colonic media did not, contrary to expectations, result in the selective enzymatic breakdown of the microspheres. Despite this unanticipated result, coated microspheres prepared at a concentration of 2.5% (w/v) CaCl_2_ were able to change drug release behavior by controlling pH and transit time. This allowed the coated microspheres to avoid drug release in the stomach and instead reach the colon in less than 24 hours to release 100% of the drug [45].

In one of the studies, 5-aminosalicylic acid (5-ASA) was compression coated with chitosan acetate (spray-dried) and ethylcellulose. The swelling behavior of the tablet compressed coated with chitosan acetate alone and with a mixture of ethylcellulose/chitosan acetate in various GI fluids (0.1 N HCl, PBS pH 6.8) and acetate buffer (pH 5.0) was evaluated. The effect of various parameters such as coating polymer ratio, stirring speed of paddle, pH of dissolution medium, caloric enzyme, and use of super disintegrant on drug release was evaluated. The results revealed that both the polymers, i.e., ethylcellulose and chitosan acetate, in the ratio of 87.5% and 12.5% provided no drug release till the first five hours and about 90% drug release within 12 hours. This system was found to be controlled (due to swelling of ethylcellulose and diffusion of the drug from its matrix) as well as pH-dependent due to the disintegration of chitosan acetate at higher pH that allowed complete drug release [46].

Sulfasalazine-loaded calcium-alginate-N-O-carboxymethyl Chitosan (NOCC) beads were formulated and coated with chitosan to target the drug to the colon. Drug (1% w/v) was added to the alginate solution, previously prepared by adding calcium-alginate-NOCC beads to a 1.5% (w/v) solution of chitosan. Swelling properties, drug content, entrapment efficiency, and drug release were studied for the beads. The swelling degree of air and freeze-dried beads at pH 1.2 was considerably low compared to the swelling degree at pH 6.8. The swelling degree at pH 1.2 and pH 6.8 was 2.8 and 46, respectively. The encapsulation efficiency of uncoated alginate beads was relatively higher, i.e., 65%, compared to coated-chitosan beads, i.e., 60%. The drug release was less in acidic pH due to less solubility of the drug in acidic media. Burst release was not observed when the formulation was introduced to alkaline media. Coating with chitosan reduced the drug release in acidic and small intestine fluid, i.e., only 40% of the encapsulated drug was found to be released after five hours [47].

It was revealed by Jain et al. that oxaliplatin is a cytotoxic agent that can be utilized for the treatment of colorectal cancer in conjunction with 5-FU and leucovorin by intravenous route. Oral administration of oxaliplatin-containing chitosan nanoparticles that had been linked with hyaluronic acid and encapsulated with Eudragit S100 was performed on tumor-bearing mice. This novel delivery system demonstrated relatively high concentrations of the drug in colonic tumor mass with a prolonged residence period, which carries the possibility of greater efficacy with decreased toxicity [48].

Kotagale et al. formulated azathioprine tablets by direct compression method using 25% of inulin polysaccharide and coated with a polymeric solution of Eudragit-S, Eudragit-L, and cellulose acetate phthalate for OCTDDS. These coating materials restricted the drug release in the upper part of GIT and released the drug in the colon in the presence of bacterial enzymes, which caused the degradation of drug coating for drug release to occur. The use of a pH modifier such as citric acid alters the pH and influences the azathioprine release from the coated formulation, which also helps to deliver the drug in the colon [49].

Ramasamy et al. reported an OCTDDS for aceclofenac that was formulated using xanthan gum as a carrier. Higher polymer concentration increased the thickness of the drug layer which lowered the release of the drug. The in-vitro dissolution study indicated that multilayer-coated tablets were degraded in the colon due to the existence of bacterial colonic enzymes, and Eudragit coating restricted the drug release in the upper part of the GIT [50].

A heterogeneous polysaccharide known as pectin is mostly made up of galacturonic acid and its methyl ester. Pectin can be found in fruits and vegetables. These are resistant to being broken down by the enzymes found in the stomach and intestines, but the enzymes produced by colonic bacteria are virtually completely capable of converting them into a chain of soluble oligo galacturonates. Pectin was used in this study as a microbially degradable polymeric carrier for the levetiracetam sustained-release tablet that was coated with Eudragit polymers. The tablet was designed to specifically target the colon and release the drug over a prolonged period of time. According to the findings of the in-vitro dissolution investigation, at the end of eight hours, 103% of the drug was released as a result of the drug’s breakdown caused by colonic bacteria in the presence of rat caecal content. The finished formulation was able to exert control over the drug release in both the stomach and the small intestine [51].

According to the findings of Niranjan et al., polysaccharide coatings such as guar gum and xanthan gum combination were utilized in microflora-triggered colon medication delivery systems. To make the compressed-coated metronidazole, guar gum and xanthan gum were utilized, both of which are employed in the treatment of amoebiasis. The in-vitro dissolution investigation showed that it inhibited the drug's release in the upper GIT and allowed for the release of 84.8 1.22% of the drug in the colon while it was in the presence of the human fecal medium [52].

Chitosan, xanthan gum, and guar gum are the three types of polysaccharides that are utilized as carriers in the process of preparing a colon-targeted drug delivery system. Tablets of metoprolol succinate were manufactured using the polysaccharides described above as binders. The tablets were then coated with Kollicoat MAE 100 DP to restrict drug release in the stomach and small intestine. The in-vitro investigation showed that chitosan releases 12.5% more of the drug in the first five hours than xanthan gum or guar gum. This was compared to the other two gums. The produced formulation showed a high level of specificity at the site of the colon, and it was found to retard the level of drug release that occurs in the colon prior to the occurrence of microbial degradation or polymer solubilization [53].

The liquisolid method was used to make the tablets that Elkhodairy et al. used to target the colon with indomethacin. The indomethacin-loaded tablets were manufactured by combining guar gum, pectin, and chitosan polysaccharides in varying amounts during the manufacturing process. To control how much of the drug is absorbed in the stomach and the small intestine, the tablet was coated with a polymer called Eudragit RL 100. According to the findings of the in-vitro dissolving investigation, the pectin-based formulation was able to release the drug for a period of 16 hours, which is the amount of time that a solid dosage form spends residing in the colon when exposed to rat caecal content [54].

In another study, colon targeting was found to be useful for the delivery of anticancer drugs such as capecitabine. Capecitabine is a prodrug that is enzymatically transformed to 5-FU (antimetabolite) by thymidine phosphorylase in cancerous cells. Once there, it inhibits DNA synthesis and halts the progress of colon tumor tissues. Compression-coated tablets were manufactured employing moringa gum as a carrier relying on a microbially induced technique for targeting colon cancer, thereby decreasing side effects associated with the oral route. The primary objective of this formulation is to concentrate the drug in the large intestine while simultaneously reducing the quantity of drug that is absorbed in the upper GIT. The tablets had a coating of HPMC K100M polymer on their surface. According to the findings of the in-vitro dissolution investigation, the produced formulation inhibited the release of the drug in the upper GIT, but the drug was fully released after it reached the colon [55].

In the study by Tiwari et al., tegaserod maleate-coated tablets were made, tested, and used for the treatment of irritable bowel syndrome. The tablets were prepared using the direct compression method. The coating on the tablets was created by combining galactosyl, xanthan gum, and chitosan polysaccharide in varying amounts. The in-vitro dissolution investigation found that when compared to galactose and chitosan, xanthan gum demonstrated more sustained drug release due to enzymatic breakdown in the presence of rat caecal material [56].

Further, the biodegradable polysaccharide dextrin was used to develop the colon-targeted azathioprine microspheres. The formulation was prepared using the solvent evaporation technique and characterized by particle size, percentage yield, drug entrapment efficiency, and surface morphology, and in vitro drug release study. Dextrin acts as a carrier that prevents the drug release in the upper GIT and releases more amount of the drug specifically in the colon. The in-vitro dissolution study revealed that 95-99% of azathioprine was released in the colonic fluid and produced local action [57].

The OCTDDS was designed for use with biologically active peptides or proteins in an effort to increase their bioavailability. In this particular research project, a multiunit nanofiber mat was produced using coaxial electrospinning. The possibility of using this mat in a colon-targeted delivery system for a bioactive peptide called salmon calcitonin was investigated. In this, the calcitonin from salmon was covered in a layer of pectin, which is a polysaccharide. The results of the in-vitro dissolution investigation demonstrated that the drug was released from the encapsulated salmon calcitonin into the colon [58].

Some of the studies pertaining to polysaccharides-based colon-targeted delivery systems are listed in Table 3.

**Table 3:** Various polysaccharide-based colon-targeted delivery systems prepared so far using different polysaccharides. 5-FU = fluorouracil; GIT = gastrointestinal tract; PBS = phosphate-buffered saline; IBD = inflammatory bowel disease

Polysaccharide	Dosage form	Description	References
Dextran	Microsphere	An azathioprine microsphere released 95-99% drug in the colonic fluid. Eudragit-coated dextran microspheres of 5-FU restrict drug release in the upper part of GIT. Enteric-coated epichlorohydrin cross-linked dextran microspheres release the drug in the colon in the presence of dextranase	Gonekar and Kori 2019; Panigrahi et al. 2012; Rai et al. 2016 [57,59,60]
	Matrix tablet	Budesonide-release 10% drug in 0.1 N HCl and PBS 7.4. The drug release increased in presence of rat caecal content in PBS pH 6.8. Albendazole matrix tablet released 96-98% drug in the colon. Matrix tablets of ibuprofen released 95-98% of the drug in the stimulated colonic fluid	Ahmadi et al. 2011; Sehabrao et al. 2009; Salunkhe and Kulkarni 2008 [61-63]
	Capsule	Glutaraldehyde cross-linked dextrans loaded with hydrocortisone at initial three-hour drug release was 10%, and at 24 hours drug the release was 35%	Brondsted et al. 1998 [64]
	Microcapsule	Budesonide microcapsule in comparison with mesalamine suspension target more drug in the colon	Varshosaz et al. 2011 [65]
	Nanoparticles	Doxorubicin nanoparticles formulation blocks tumor growth in colorectal carcinoma	Li et al. 2015 [66]
	Hydrogel	Hydrocortisone hydrogel was prepared based on dextran cross-linked with diisocyanate showed higher degradation of the drug in the colon	Hovgaard and Brondsted 1995 [67]
Guar gum	Matrix tablet	Guar gum-based matrix tablets of rofecoxib release 5-12% of the drug in the upper GIT and 57% of rofecoxib in colonic fluids. A colon-specific mebendazole which contains 20% guar gum released 80% of the drug and another contains 30% of guar gum which released 50% of the drug in the colonic fluid. Albendazole released 44% of the drug in the stimulated colonic fluid. Metronidazole reduced the swelling and premature drug release in the upper GIT. Guar gum-coated mesalazine matrix tablets protect the drug release in the stomach and small intestine due to their high viscosity. The guar gum and starch paste containing mesalamine matrix tablets improve the bioavailability and colon-specific release of the drug for the treatment of ulcerative colitis. Tinidazole tablet showed delayed Tmax, decreased Cmax, and Ka indicated that the drug is not released in the upper part of the GIT and is available in the colon to produce a local effect. After the 20-hour period, the 5-ASA tablet began to release the drug indicating the disintegration of the coating by caecal enzymes. The in-vitro dissolution studies revealed that piroxacam tablets increase drug release in the presence of rat caecal content. Metronidazole tablets decrease the drug release in the first five hours and release 61% of metronidazole in the colon	Krishnaiah et al. 2001; Al-Saidan et al. 2005; Krishnaiah et al. 2001; Krishnaiah et al. 2002; Krishnaiah et al. 2002; Tugsu et al. 2004; Shinkar and Dehghan 2014; Krishnaiah 2003; Chaurasia et al. 2006; Veerareddy and Manthri 2010 [3,38,40-42,68-72]
	Microsphere	Cross-linked guar gum microsphere of 5-FU delayed the apoptosis and produced the sustained release of the drug in colon cancer. Guar gum cross-linked with glutaraldehyde microspheres loaded with methotrexate release the drug in the colon to produce the local effect in the colon	Kaushik et al. 2009; Chaurasia et al. 2006 [30,71]
	Nanoparticles	The methotrexate-loaded folic acid conjugated nanoparticles protect the drug release in the upper part of the GIT and produced the local effect in the treatment of colorectal carcinoma	Sharma et al. 2013 [73]
	Spheroids	Sulfasalazine spheroids showed significantly higher drug release in the colon. The in-vivo studies performed on rats showed the therapeutic advantage of co-administration of probiotics with guar gum	Prudhviraj et al. 2015 [74]
	Granules	5-FU granules coated with guar gum and Eudragit FS30D released 91.61% of the drug in probiotic culture medium	Kotla et al. 2016 [75]
	Microparticles	Microparticles of doxorubicin and metformin hydrochloride released the drug in the stimulated colonic fluid to produce a local effect for the treatment of cancer	Kang et al. 2020 [76]
	Immediate release pellets	Guar gum-lactose-hydroxypropyl methylcellulose-coated indomethacin immediate-release pellets loaded in pulsatile capsules showed higher efficacy in colon targeting	Yang et al. 2020 [77]
	Microcapsule	The tinidazole-loaded guar gum microcapsule was prepared and evaluated to produce a local effect in the colon for the treatment of amoebiasis	Debnath et al. 2013 [78]
Xanthan gum	Multilayer-coated tablets	Aceclofenac restricts the drug release in the upper part of the GIT. Tegaserod maleate-coated tablets showed better-controlled drug release due to enzymatic degradation in the presence of rat caecal content	Ramasamy et al. 2011; Tiwari et al. 2018 [50,56]
	Matrix tablets	Metronidazole enhanced the drug release in the presence of rat caecal content for colon targeting. Methotrexate tablets deliver the drug at the colonic site	Niranjan et al. 2013 [52]
	Nanoparticles	5-FU nanoparticles were evaluated in rat “caecal content.” The result of the studies indicated that the microflora present in the GIT was damaged due to the administration of 5-FU	Singh et al. 2015 [79]
Chitosan	Matrix tablets	The chitosan releases 12.5% metoprolol succinate in the first five hours and high specificity at the site of the colon. Mesalamine tablets along with chitosan showed 43% which meant the drug release was inhibited. Metronidazole releases 80% of the drug in the colon	Godge and Hiremath 2014; Patel et al. 2009; Mehta et al. 2011 [53,80,81]
	Microspheres	Mesalamine microspheres restrict the drug release in the stimulated gastric fluid. The formulated microspheres of albendazole show 10-fold increases in the bioavailability of albendazole. 5-FU microspheres showed 80% of drug release in the colon	Badhana et al. 2013; Garcia et al. 2015; Shivani et al. 2011 [82-84]
	Beads	Albendazole showed excellent sustained-release activity which could be further developed into the treatment of gastrointestinal disease. Chitosan hydrogel beads formulated for the delivery of satranidazole to the colon	Wang et al. 2010; Jain et al. 2007 [85,86]
	Microcapsules	Albendazole showed maximum drug release in rat caecal content at the end of 24 hours. Tinidazole chitosan microcapsules showed slower drug release. In-vitro drug release in the dissolution medium containing rat caecal content was higher	Simi et al. 2010 [87]
	Nanoparticles	Metronidazole showed controlled drug release over a period of 12 hours. 5-ASA nanoparticles showed the site-specific release of the drug at the site of the colon for the treatment of IBD	Elzatahry and Eldin 2008; Markam and Bajpai 2020 [88,89]
	Mucoadhesive hydrogels	Sulfasalazine decreased residence colonic time. Tinidazole and theophylline showed drug release kinetics was in accordance with the Korsmeyer-Peppas model	Xu et al. 2017 [90]
	Microparticles	Albendazole formulation showed a marked increase in the oral bioavailability of the drug. Albendazole-chitosan microparticles showed a remarkable increase in the dissolution profile of the loaded drug in comparison to the release profile of pure API	Piccirilli et al. 2014; Leonardi et al. 2008 [91,92]
Pectin	Tablet	Indomethacin tablets release the drug for 16 hours, which is the residence time of a solid dosage form in the colon in the presence of rat caecal content. Pectin and ethylcellulose combination to prepare osmotically controlled tablets on paracetamol used to treat colonic-related diseases. Ropivacaine tablets increased the release of the drug in the stimulated colonic fluid. Rhubarb (herbal drug) showed site-specific release. In-vitro release kinetic showed zero-order release of resveratrol-loaded matrix tablets	Elkhodairy et al. 2014; Wakerly et al. 1997; Gandhi et al. 2020 [54,93,94]
	Multi-unit nanofiber mat	Coaxial electrospinning manufactured a multi-unit nanofiber mat of salmon calcitonin showed released the drug in the colonic environment	Feng et al. 2019 [58]
	Microsphere	Metronidazole microsphere coated with Eudragit S-100 releases the drug in the presence of rat caecal content. Metronidazole prodrug-bearing microspheres restrict the drug release in acidic pH and release in the colon. 5-FU microspheres released 66.32% of the drug in the colon	Vaidya et al. 2015; Vaidya et al. 2009; Paharia et al. 2007 [95-97]
	Microparticles	Ketoprofen release was retarded due to zinc cross-linking with pectin	El-Gibaly 2002 [98]
	Nanoparticles	5-FU nanoparticles targeting the HT-29 colon cancer cells. Resveratrol nanoparticles controlled the delivery of the drug at the site of the colon	Subudhi et al. 2015; Prezotti et al. 2020 [99,100]
	Beads	Metronidazole beads showed high drug entrapment. With this, sustained release of water-soluble drugs was achieved. Tinidazole microbeads showed 70.83% of drug release in 24 hours in the colon	Pawar et al. 2008 [101]
Chondroitin sulfate	Matrix tablets	The degradation of chondroitin sulfate by bacterial enzymes released aceclofenac in the simulated colonic fluid. Indomethacin-loaded chondroitin sulfate tablets improved the delivery of the drug at the site of the colonic milieu	Ramasamy et al. 2012; Amrutkar and Gattani 2009 [28,102]
Inulin	Tablet	Azathioprine restricts the drug release in the upper part of the GIT and releases the drug in the colon in the presence of bacterial enzymes	Kotagali et al. 2010 [49]
	Hydrogel	Cinnamate hydrogel showed site-specific release in the colon	Lopez-Molina et al. 2015 [103]
Ethylcellulose	Pellets	Pellets released 85% of 5-FU in stimulated colonic fluid	Wei et al. 2007 [104]
	Tablets	Dapsone tablets controlled the drug released at the site of the colon	Barros et al. 2020 [105]
Alginic acid	Microbeads	The formulated 5-FU microbeads were found to be biodegradable and non-toxic. The in-vitro study revealed that 5-FU beads 90% of the drug released in the presence of colonic enzymes	Sun et al. 2019; Agarwal et al. 2015 [106,107]
Hyaluronic acid	Nanoparticles	Oxaliplatin nanoparticles showed higher local concentrations at the site of the colon for the treatment of colon cancer. Budesonide nanoparticles showed targeted drug delivery for the treatment of IBD. 5-FU nanoparticles showed apoptosis in colon cancer cells. Camptothecin and curcumin-loaded nanoparticles showed a sustained-release profile of both drugs for the treatment of colon cancer. Hyaluronic acid-loaded irinotecan nanoparticles formulated for monitoring and the treatment of colon cancer	Jain et al. 2010; Vafaei et al. 2016; Liu et al. 2015; Xiao et al. 2015; Choi et al. 2012 [48,108-111]

Dissolution methods used for evaluating polysaccharide-based OCTDDS

A very popular triggering mechanism exists which is the fermentation of non-starch polysaccharides by the colonic microflora to gain the delivery of drug in the colon, which is devoid or dependent on GIT transit time, pH, and disease status. Because the colon consists of numerous colonies of microorganisms that are unique and diverse in their orientation, it possesses a huge challenge for the development of a USP dissolution medium and scale-up and technology transfer for this technology [4].

Dissolution Medium Using Rat Caecal Contents

Rat caecal media was developed because rats were readily available, and traditional USP dissolution media was the dominant form of dissolving media at the time. In addition to this, they had the same microbiota as humans. Bacteroides and Bifidobacteria colonies are typically found to populate the caecal medium. Just prior to dissolution, the production of this medium begins to preserve the anaerobic conditions that are characteristic of the rat caecum. PBS solution with a pH of 7 is typically used to dilute it before use. To maintain anaerobic conditions, the step before this one is typically carried out in an atmosphere containing carbon dioxide. Executed in a dissolution bowl at a temperature of 37°C, after which the bioanalytical technique development for HPLC is carried out [43,112].

In 1993, Rubinstein and colleagues investigated the rate at which the medication indomethacin was released from calcium pectinate tablets in 100 mL of PBS at a pH of 7.0 and in the absence of 1.25% (w/v) of rat caecal contents. Based on the findings, it was deduced that the amount of indomethacin that was released was much higher in concentration when compared to the standard control.

In 1998, Prasad and colleagues investigated how the concentration of rat caecal contents affected drug release. This was evaluated in a guar gum matrix formulation using indomethacin as the drug under research in USP dissolve apparatus I. This was investigated simultaneously with the contents of rat intestines. The analysis of the data consisted of taking the mean of three separate studies under stirring at 100 revolutions per minute (rpm), at 37°C, the dissolution was carried out in a beaker with a capacity of 150 mL containing 100 mL of PBS with a pH of 6.8. The beaker was continually sparged with carbon dioxide to ensure that anaerobic conditions were maintained. Before collecting the caecal contents to facilitate the proliferation and activity of guar gum-degrading bacteria, the rats were given 1 mL 2% (w/v) guar gum to eat for zero, three, and seven days. The presence of this caecal content resulted in a three- to four-fold increase in drug release showing the likelihood of colon-specific drug release [36].

Chang et al. in 2019 studied rhubarb as a drug and its effects. The stirring speed of 75 rpm was used at a temperature of 37°C in pH 7.4 buffer containing rat caecal contents at 0.4% SDS having nitrogen gas continuously pumped through it. It was reported that this dissolution test was reproducible [113].

Dissolution Medium Using Human Fecal Slurry

Slurries made from human feces have been utilized to great effect in research attempting to determine the fermentation of non-starch polysaccharides. Because it comprises 55% of fecal particles, the generation of simulated colonic fluid (SCF) required that acetate, propionate, and butyrate be evaluated for their function as a function of fermentation duration. When researching the fermentation of non-starch polysaccharides, fresh slurries of human feces are typically the medium of choice. In most cases, the solutions for this study are made by homogenizing fresh feces that have been taken from healthy persons at a concentration of 5% (w/v). After that, these contents are put through an alkaline test, and conditions are kept stable by purging the atmosphere with carbon dioxide.

The release of 5-ASA from pellets that had a coating with amylose and ethylcellulose of varied ratios was investigated using human fecal slurries at a concentration of 5% (w/v). When preparing slurries of human feces for homogenization in anaerobic settings, PBS with a pH of 7 was utilized as the dispersant of choice. It was verified that the donors had not taken any antibiotics in the three months leading up to the experiment, as that was a prerequisite for participation. Samples of liquid and gas head space were taken from the fermenter at a rate that was previously defined and kept constant for a period of 48 hours. Gases including hydrogen and carbon dioxide, as well as volatile fatty acids, were used in the analysis of the level of fermentation. These liquid samples were centrifuged to get rid of microorganisms and to make room for a subsequent HPLC examination that took place in freezing conditions. The ratio of 1:4 for amylose/ethylcellulose 5-ASA was totally released in a period of time that lasted for six hours. The entire procedure was carried out in a basket containing pH 1.2 and pH 7.2 buffers, and it had a capacity of 900 mL. When fermentation was allowed to continue for a longer period of time, a higher concentration of volatile fatty acids was produced, which was evidence that amylose was destroyed in the coated film [114].

In the study by Zhang and colleagues in 2019, four human healthy volunteers (two males and two females) provided fresh fecal samples. A screening was done to ensure that they had not had any antibiotic medication or suffered from any gastrointestinal condition in the three months prior to the collection. To create a slurry with a concentration of 10% (w/v), the fecal samples were first diluted in 0.9% (w/v) saline. After the fecal slurries had been collected in jars, they were kept in anaerobic conditions before being centrifuged at 500 g for five minutes. This slurry was injected into a media that already had 1 mL of fecal suspension mixed into 9 mL of basic nutrition media. In a thermostatically controlled shaker, each of the treatment groups was given an incubation period at a temperature of 37°C. Conditions similar to anaerobic respiration were preserved throughout this period. After zero, six, 12, 24, and 48 hours of fermentation, the samples were put through additional testing. This study provided more support for the hypothesis that certain oligosaccharides were not digested [115].

Dissolution Medium Using Goat Caecal Contents

Goat caecal contents are easy to procure as they are easily available in local slaughterhouses. They share the same physiology and environment as the human gut. Hence, the incorporation of goat cecum contents for in-vitro dissolution is an easier and more economical process.

In 2012, Ahmad et al. conducted a dissolution study using goat caecal contents that were obtained from a local slaughterhouse. The goat was stored in a physiological solution prior to the experiment. This test was performed in a USP type I dissolution media where the stirring speed was maintained at 100 rpm and the temperature was kept at 37°C. The release of metronidazole from the microspheres was carried out in 0.1 N HCl in the first two hours, followed by pH 7.4 PBS. Anaerobic conditions were maintained by continuously bubbling CO_2_ in the dissolution medium. The samples were subjected to HPLC analysis. This study was performed in triplicate. The release of the drug in simulated gastric fluid and simulated intestinal fluid was insignificant (p < 0.001) [116].

Dissolution Medium Containing Prebiotic/Probiotic Culture

A method that was able to simulate colonic fluid was one in which a mixture of five probiotics was produced and carried out in the presence of a prebiotic while the process was carried out in anaerobic conditions.

In a study conducted in 2014 by Fares and colleagues, compressed curcumin inulin nanoparticles were introduced into a USP-dissolving type 2 device in the shape of tablets. This was stored in a cylinder with a capacity of 900 mL and a pH 5.5 buffer at a temperature of 37°C. The speed of the stirrer was set to 100 rpm. Five milliliters of the material were removed and replaced. A membrane filter was utilized to purify it. The proteolytic equilibrium of the curcumin was altered as a result of the change in pH. When the concentration of the drug changes, so does the pattern of how it is released. The point of inflection of nanoparticles that are generated at pH 7.0 indicates a drop in the k value. This demonstrates that the intermolecular interactions between inulin and curcumin are at their optimum level. Because of this, the n value ends up being quite high at 0.53, which makes it easier for curcumin to be released. It was decided that this would be the most effective release at pH 7.0 [117].

A study of spheroids to target the colon was conducted by Singh et al. in 2015 where they used three different media. To maximize the amount of medication that was liberated from sulphasalazine spheroids, the gradient pH-dissolving approach was utilized. A USP dissolution equipment I was utilized so that this could occur. The temperature was held steady at 37°C throughout. The first two hours of the experiment were carried out with a stirring speed of 100 rpm in 200 mL of an HCl solution having a pH of 1.2. A CO_2_ gas purge was performed on the dissolving medium after the volume was brought up to 900 mL and the pH was brought down to 7.4. The capacity was brought up to 1,000 mL by adding more fluid thioglycollate medium (FTM) to the probiotic culture that was initially 100 mL. Five milliliters of samples were removed and replaced. The percentage cumulative release of sulphasalazine from spheroids in FTM containing probiotic culture (procured BIOMIX culture) exhibited a release of 93% in eight hours, which was greater than the amount that was released into the negative control media [118].

Enzyme-Triggered Dissolution Method

This model is based on the idea that enzymes that are produced naturally by the colonic microflora digest the polysaccharide that is either in the coating or in the matrix facilitating the drug release. This process calls for the utilization of a wide variety of enzymes, some of which are galactomannanase, amylase, pectinase, and chondroitinase. The following factors contribute to why this approach does not demonstrate prominence in vivo [118]: the enzyme concentration used in the media in vitro decreases in a very predictable manner as this is responsible for the degradation of the polysaccharides; the susceptibility of the polysaccharide to one enzyme tends to change; and in the dissolution media there is only one specific media as opposed to a wide variety that is present in the colon.

Philip et al. in 2008 formulated galactomannanase to study capsules of the drug cefadroxil. It delayed the release of the drug in the stomach for two hours and then showed controlled release in the media that corresponded to the intestine. This was different from the agitational intensity and the defects that were caused by the release membrane [119].

Lai et al. in 2010 prepared compression-coated tablets of lansoprazole using guar gum as the polymer for colon targeting. In-vitro studies were performed in a series of dilutions that contained β-mannanase as it could specially degrade the guar gum in this media. From the results, it was noticed that the release of the drug was faster as it was exposed to a colonic dissolution media containing β-mannanase. The release profile in SCF of humans and rats was dissimilar when compared to the enzyme-linked dissolution study and could not substitute for the same. It was noticed that β-mannanase could hydrolyze the plant gums used in the formulation [120].

**Figure 2:**
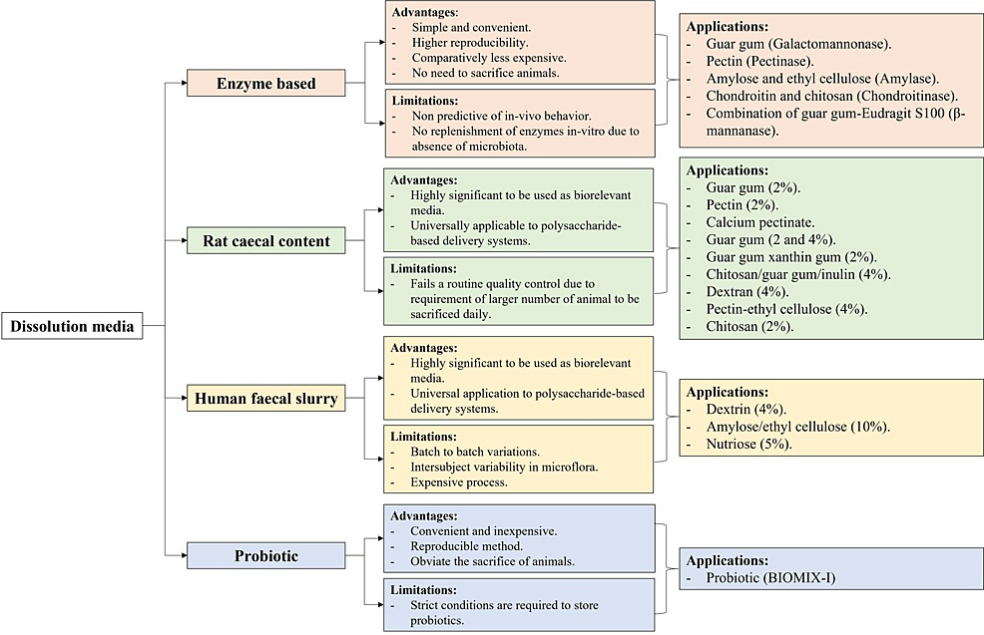
Approaches used for the dissolution testing of polysaccharide-based OCTDDS. The figure was created by the author to summarize the information in the last section. OCTDDS = oral colon targeted drug delivery system

Figure 2 summarizes the aforementioned dissolution testing methods used to evaluate the drug release from polysaccharide-based OCTDDS.

## Conclusions

The development of OCTDDS has always been a consistent challenge for formulation scientists due to the highly dynamic environment of the GIT. Most marketed formulations are in delayed-release form due to certain inherent limitations such as the change in pH of the GIT during the diseased state, the selection of specific enzymes to act on the prodrug, and the lack of microflora during the diseased state. Among all reported approaches, the polysaccharide-based approach has been found to be the most suitable to successfully target drugs to the colon. Moreover, excellent articles have been published in the past two decades in the area of the development of polysaccharide-based OCTDDSs, wherein drug release has been significantly restricted to less than even 10% in the first five hours, followed by either sustained-release profile or burst-release profile. Even the pharmacokinetic studies of those research works have shown a delayed absorption of the drug after five hours. Despite this, none of the polysaccharide-based formulations reach the clinical level because this picture of delayed-drug release looks good in a healthy subject having a plethora of microbes to digest the polysaccharide matrix/coat. However, during the diseased state, the count of the microbiota is significantly decreased, and, due to this, there remains a scarcity of bacteria in the colon to digest the polysaccharide. Overall, the formulations may come out with partial or no release out of the body.

To overcome such challenges the co-administration of synbiotics along with polysaccharide-based formulation has been introduced by researching colitis-induced rats. The proposed hypothesis is based on the fact that the co-administration of probiotics would compensate for the bacterial loss, and on subsequent dosing, it will also help in the multiplication of bacteria in the body. Furthermore, it would also help in treating the disease. This research is still in its initial stages and tremendous efforts are required to gather more results by conducting clinical studies. In time to come, the polysaccharide-based delivery system co-administered with synbiotics will provide a promising future to scientists working in the area of colorectal diseases.
